# Case of Bite of a Young Woman by a Large Cobra De Capello

**Published:** 1853-05

**Authors:** Wm. Chalmers

**Affiliations:** Lately Surgeon H.E.I.C. service, Bengal


*Case of Bite of a Young Woman by a large Cobra de Capello.* By Wm. Chalmers, M.D., lately Surgeon H.E.I.C. service, Bengal.—On 25th of June, 1819, at 11 p. m., I heard from the outside of my house, at Barrackpore, near Calcutta, a loud call for my immediate attendance. It proved to be from Colonel, afterwards General Sir Wm. Lumley, whom I found with a lanthorn in his hand, entreating me for God's sake to come with him at once, as his *mehturanee* (female sweeper) had been bitten by a cobra de capello. I took in my hand a phial of solution of ammonia, of the usual strength, a case of scalpels, and a large sized elastic gum male catheter. On arriving at the hut occupied by the poor woman and her husband, I found her stretched outside on the ground, her head resting on her husband's knee. Her body was cold and collapsed; there was neither breathing nor pulse; her eyes were wide open, and insensible to light; the mouth was also wide open; tongue cold; in fact life was, to all appearance, extinct. How long she had lain in this position could not be ascertained; her husband conjectured an hour at least. On the back of the right hand were discovered two punctures, as if made by a needle, about an inch and a-half apart, marking the entrance of the poisonous fangs of the snake. Upon each puncture there was a drop of nearly colorless fluid, without any hæmorrhage, tumefaction, or ecchymosis.

Here was a case sufficiently discouraging, if not, to all human appearance, hopeless. However I resolved not to abandon the poor sufferer, while the kind-hearted master entreated me to do what I could.

Ordering bricks to be heated for application to the præcordia and the feet, the first step of the treatment was to pour down her throat a teaspoonful of the ammonia, with as much water, but all power of deglutition being lost, some difficulty was experienced in accomplishing my object. By the aid, however, of the catheter as an œsophagus tube I succeeded admirably. The next step was to cut out and pare off the integuments and subjacent cellular and muscular tissues, extending my incision about one-fourth of an inch beyond the punctures. From the large wound, which was of an oval shape, not a drop of blood escaped in this operation. The husband was now directed to apply his mouth to the wound, and suck with all his powers, which he proceeded to do most readily, the natives having great faith in such a measure. This he continued, with all the energy he was capable of, for fully half-an-hour, without succeeding in procuring any moisture, while I repeated the ammonia steadily every ten minutes, till a full ounce was consumed. At length our perseverance was rewarded by some hopes of a restoration, for the poor distracted husband leaped up in extasy of joy, exclaiming in his own language, "*Kohoo àtá sahib*," (blood is coming, Sir), showing his tongue covered with the vital fluid. In a few minutes more the action of the heart was faintly perceptible; the pulse at the wrist was just traceable in a thready thrill; she moved her head, gave a deep sigh and sat up. Thus our persevering efforts for nearly two hours, were rewarded by the rescue of a fine young healthy woman from certain death;—in truth her recovery might be considered, without any hyperbole, a resurrection from the dead. The only treatment pursued afterwards was the free cauterization of the wound by *Nitras Argenti*, —the application of a pledget of lint dipped in melted *Ceratum Resinæ*, covering the whole with a hot poultice. The wound healed kindly by granulation, and she was able to resume her duties in a few days.

In this remarkable case the powers of the ammonia were proved most incontestably as a safe and sure remedy, as it has ever since and during my time in India—extending now to a quarter of a century—been esteemed and experienced to be, in all bites of venomous reptiles. Indeed, such is the confidence of the natives in the remedy, and more especially the native soldiery, who in their huts are much exposed to these injuries, that the moment any one gets bitten, he runs to the nearest European officer, calling out for the *Safed Dâwy*, "the white medicine," assured that with a few doses of that, he is safe, and disappointment in the curative effects is never experienced. By "the white medicine" is meant a preparation of ammonia, which used to be imported in great quantities from England, and generally kept at hand by every officer,—the old fashioned *Eau de Luce, Liquor Ammoniæ Succinatus.*

One cannot help regretting, that the medical officers of the hospital into which that poor unfortunate fellow, who was bitten in the Zoological Gardens last year, was admitted, were not apparently sufficiently acquainted with the powers of ammonia in such cases. If they had been, it would no doubt have been had recourse to.—*Glasgow Medical Journal*, April, 1853.

